# Anticancer potentiality of lignan rich fraction of six Flaxseed cultivars

**DOI:** 10.1038/s41598-017-18944-0

**Published:** 2018-01-11

**Authors:** Shahira M. Ezzat, Samia A. Shouman, Abeer Elkhoely, Yasmin M. Attia, Mohamed S. Elsesy, Amira S. El Senousy, Mouchira A. Choucry, Sabah H. El Gayed, Abeer A. El Sayed, Essam Abdel Sattar, Nebal El Tanbouly

**Affiliations:** 1 Pharmacognosy Department, Faculty of Pharmacy, Cairo University, Kasr El-Einy Street, Cairo, 11562 Egypt; 2 Department of Pharmacognosy, Faculty of Pharmacy, October University for Modern Science and Arts (MSA), 12566 6th October, Egypt; 3 Cancer Biology Department, National cancer institute, Cairo University, Cairo, Egypt; 4 Pharmacology and Toxicology Department, Faculty of Pharmacy, Helwan University, Helwan, Egypt

## Abstract

The objective of our study is to highlight the therapeutic effect and mechanism of action by which purified Flaxseed hydrolysate (PFH) which is a lignan rich fraction exerts its anticancer activity on a human breast cancer cell line (T47D) and in mice bearing tumor. HPLC analysis of PFH of six flaxseed cultivars had shown that PFH of the cultivar Giza 9 (PFH-G9) contains the highest concentration of SDG (81.64 mg/g). The *in vitro* cytotoxic potentiality of PFH’s of six flaxseed cultivars was screened against a panel of human cancer cell lines. PFH -G9 showed the most significant cytotoxic activity against ER-receptor positive breast cell lines MCF7 and T47D with IC_50_ 13.8 and 15.8 µg/ml, respectively. Moreover, PFH-G9 reduced the expression of the metastasis marker, 1-α, metalloproteinases and vascular endothelial growth factor (VEGF), one of the most potent stimulators of angiogenesis, while it increased the caspase-3 dependent apoptosis. Our study also showed that dietary intake of 10% of Giza 9 Flaxseeds (FS), fixed oil (FSO) or Flax meal (FSM) twice daily for 3 weeks in mice-bearing solid Ehrlich ascites carcinoma (EAC) resulted in reducing the tumor volume, the expression of estrogen, insulin growth factor, progesterone, VEGF and MMP-2, but enhanced expression of caspase-3.

## Introduction

Given the continuous rise in cancer incidence together with the associated high mortality, in addition to the spiraling healthcare costs of treatment, there is always an interest in finding new strategies for its prevention and even treatment. In this respect, there is a substantial tendency for the use of natural compounds as anticancer or chemopreventive agents.

A thousand of studies have demonstrated the role of phytoestrogens as health promotors, and in protecting against or treating cancer^1^. Lignans and their *in vivo* metabolites, especially enterolactone (ENL) had acquired great interest as potential chemopreventive and therapeutic agent^2^. Lignans are natural plant constituents that are referred to as phytoestrogens. Phytoestrogens are plant constituents that can interfere with the estrogen metabolism. Lignans can modify the estrogen levels through their estrogenic and anti-estrogenic effect. Daily uptake of lignans as a dietary supplements may induce hormonal changes which are of high benefits to females at all ages^3,4^.

Flaxseed is the richest natural source of lignans, as its lignan content is 100 times greater than other lignan containing grains, fruits and vegetables (9–30 mg/g)^5,6^. Flaxseed lignans were shown to possess potent antitumor activity for hormone-sensitive cancers^7^. Dietary supplementation of flaxseed or its lignan precursor; secoisolariciresinol diglucoside, SDG) for two weeks prevented metastasis of B16BL6 murine melanoma cells in mice^8,9^. Moreover, 5% flaxseed in the diet inhibited the growth and development of the transgenic prostate adenocarcinoma in mice^10^. Another study has shown that the dietary intake of flaxseed in mice bearing estrogen (ER)-receptor negative human breast cancer (MDA-MB-435), reduced tumor growth and metastasis through down-regulation of insulin-like growth factor^11^. Additionally, flaxseed, its lignan and oil components were also reported to decrease mammary tumor growth at a late stage of carcinogenesis^12,13^. Also, Dietary intake of flaxseed oil which is a rich source of ω−3 fatty acid suppressed colon tumor in rats particularly in the promotion stage^14–16^. Moreover, flaxseed oil also decreased estrogen (ER) receptor-positive human breast (MCF7) tumorigensis through downregulation of the growth factor mediated pathway^17^.

The objective of this study was to introduce the Egyptian flaxseed, which is a cheap and available nutrient, as an anticancer drug. To achieve our aim a comparison between different cultivars of flaxseed cultivated in Egypt on the basis of their lignan content as well as their *in vitro* cytotoxic effect on different human cancer cell lines was performed, in addition, an *in vivo* anticancer study for the most active cultivar was conducted.

## Results

### HPLC analysis of the purified hydrolysates of the six flaxseed cultivars

**Figure 1:**
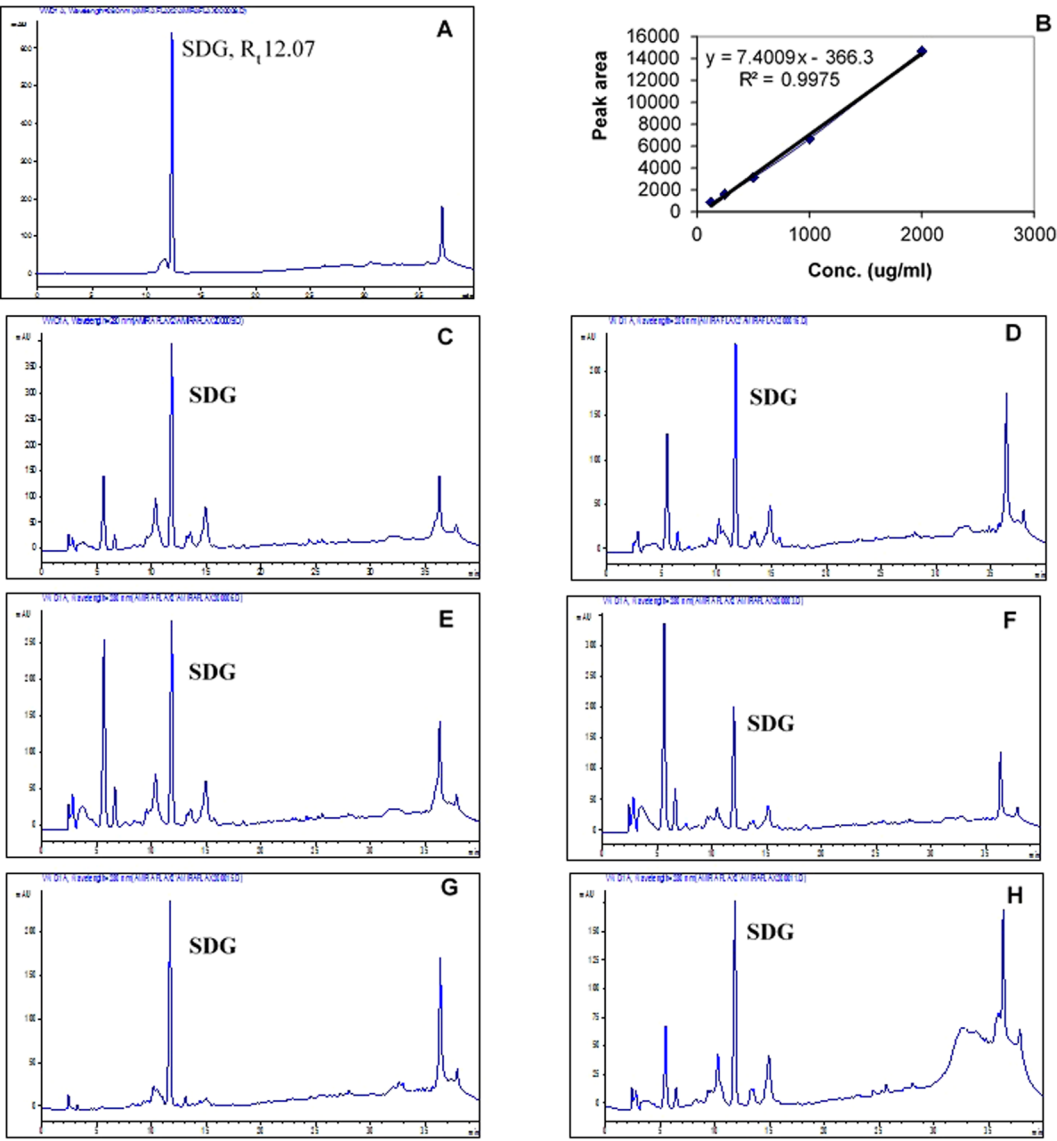
HPLC analysis of the purified hydrolysates of the six flaxseed cultivars, (**A**) chromatogram for standard SDG, (**B**) standard calibration curve for SDG, (**C**) chromatogram for Giza 9, (**D**) chromatogram for Giza 10, (**E**) chromatogram for Sakha 1, (**F**) chromatogram for Sakha 2, (**G**) chromatogram for Sakha 3, (**H**) chromatogram for Sakha 4.

HPLC analysis of the hydrolysates after being purified over a Diaion HP-20 of the six flaxseed cultivars showed that Giza 9 cultivar is the richest one in SDG as it contains 0.845 ± 0.0206 per gram hydrolysate which is equivalent to 81.64 mg/g of the total 80% methanol extract of the defatted powder (Table 1 and Fig. 1).

**Table 1:** HPLC analysis of six flaxseed cultivars.

Sample	Content (SDG gm/gm purified hydrolysate)	Methanolic Yield (gm MeOH/100 gm powder)	Content (SDG mg/gm methanolic extract)	Content (gm %) (SDG gm/100 gm methanolic extract)
Sakha 1	0.648 ± 0.0023	5.96	53.19	5.32
Sakha 2	0.487 ± 0.0127	4.61	38.6	3.9
Sakha 3	0.489 ± 0.0092	7.20	54	5.4
Sakha 4	0.437 ± 0.0036	8.60	34.5	3.45
Giza 9	0.845 ± 0.0206	10.80	81.64	8.16
Giza 10	0.505 ± 0.0007	11.65	4.43	0.43

### *In vitro* study

#### Cytotoxic screening of the PFH of the six flaxseed cultivars

Table (2) shows the IC_50_ of all the investigated hydrolysates on 5 cancer cell lines (HELA, TD47, MCF7, MDA-MB231 and HCT116 in addition to 1 normal cell line HFB4. It was detected that all the tested hydrolysates had cytotoxic activity with IC_50_ between (12.8–24.3ug/ml). All the hydrolysates showed less cytoxicity to normal cell line compared to cancer cell lines. PFH from Sakha 2, Giza 9 and Giza 10 have more significant cytotoxic activity against ER-receptor positive human breast cancer cell lines; MCF7 and T47D compared to the effect on the triple negative cell line MDA-MB231. Moderate cytotoxicity was detected by all hydrolysates on colorectal cancer cell line, HCT116. It is also worthy to mention that Sakha 1 had the least significant activity against HELA, MCF7 and T47D cell lines. On the other hand, Sakha 3 had strong activity against HELA, and T47D, while Sakha 4 showed its most pronounced activity against MCF7 and T47D.

**Table 2:** *In vitro* cytotoxic screening of the hydrolysates of the six flaxseed cultivars.

IC_50_^*^ values (ug/ml)						
	HELA	T47D	MCF7	MDA-MB231	HCT116	HFB4
PHF of Sakha 1	22.6	20.3	21.3	22.8	17.4	28.2
PHF of Sakha 2	19	12.8	19.8	24.3	18.6	36.6
PHF of Sakha 3	18.6	15.8	21.3	24.8	18.2	30.2
PHF of Sakha 4	23.8	14.3	15.8	21.8	18.6	41.4
PHF of Giza 9	17.4	15.8	13.8	23.8	24.6	24.2
PHF of Giza 10	18.6	15.3	14.8	25.3	19.8	38

*IC_50_ ± SD of 3 independent experiments performed in duplicates.

PFH of Giza 9 (PFH-G9) which showed a significant cytotoxic activity against both MCF7 and T47D cell lines was chosen for further *in vitro* investigations.

#### Effect of PFH-G9 on the expression of MMP-2 and MMP-9 in T47D cells

**Figure 2:**
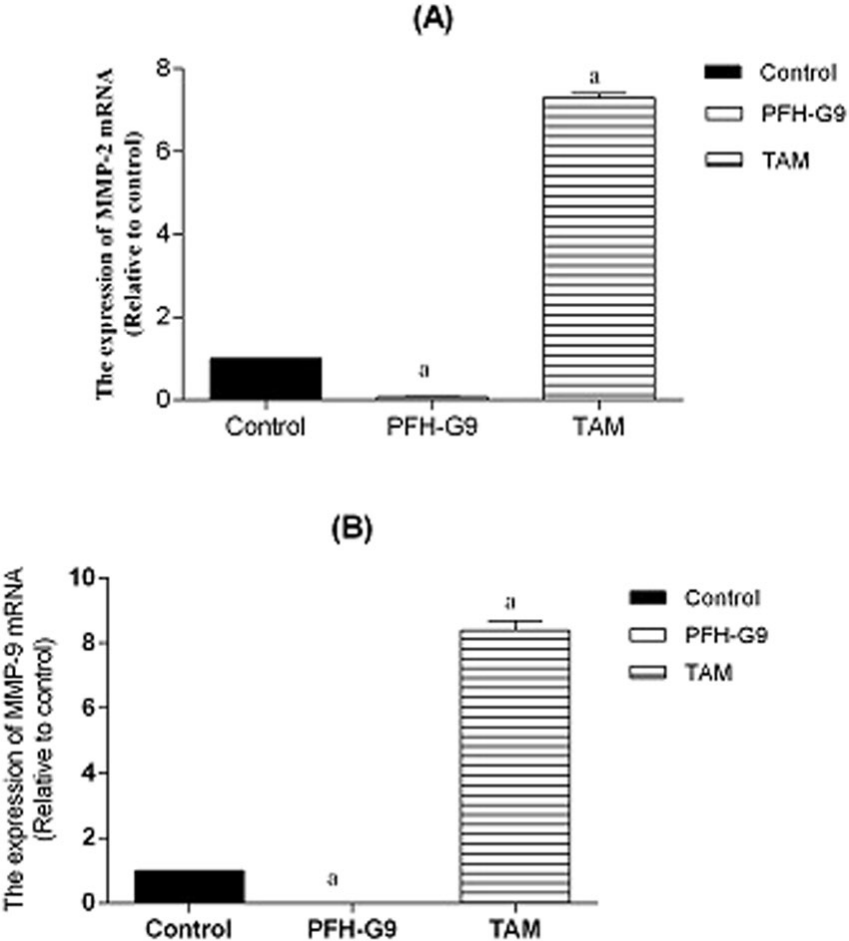
Effect of treatment of T47D cells with IC_50_ of PFH-G9 (15 μg/ml) or TAM (15 μM) for 48 h on the Expression of (**A**) MMP-2 and (**B**) MMP-9 mRNA. Each point is the mean ± SD (n = 3) a: Significantly different from control group at *p* < *0.05* using ANOVA followed by Tukey-Kramer as post-hoc test.

Figure (2) illustrates PFH-G9 at IC_50_ (15 μg/ml) decreased the expression levels of MMP-2 and MMP-9 mRNA by 92% and 99.5%, respectively compared to the control group. However, treatment of T47D cells with Tamoxifen (TAM) (15 μM) significantly resulted in 6.3-fold and 7.4-fold increase in the expression levels of MMP-2 and MMP-9 mRNA, respectively compared to the control group.

#### Effect of PFH-G9on caspase-3 activity in T47D cells

**Figure 3:**
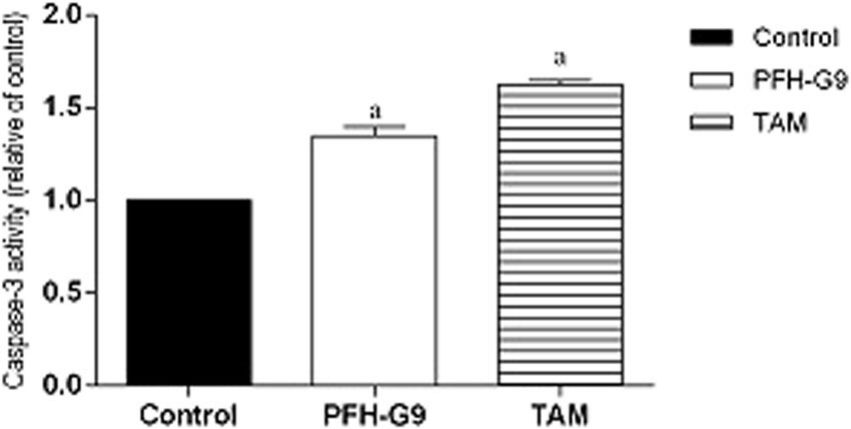
Effect of treatment of T47D cells with IC_50_ of PFH-G9 (15 μg/ml) or TAM (15 μM) for 48 h on caspase-3 activity. Each point is the mean ± SD of three independent experiments performed in duplicate. a: Significantly different from control group at *p* < *0.05* using ANOVA followed by Tukey-Kramer as post-hoc test.

To investigate the apoptotic effect of PFH-G9 on T47D cells, caspase-3 activity was assessed. PFH-G9 (15 μg/ml) induced an elevation of caspase-3 activity by 35% compared to the control group. Moreover, TAM (15 μM) significantly induced a 62.5% increase in caspase-3 activity compared to the control group, Fig. 3.

#### Effect of PFH-G9on p53 the mutated in T47D cells

**Figure 4:**
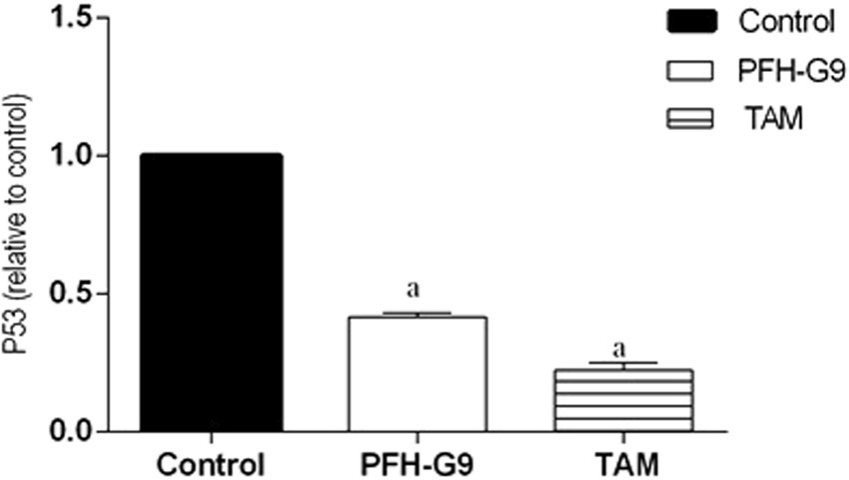
Effect of treatment of T47D cells with IC_50_ of PFH-G9 (15 μg/ml) or TAM (15 μM) for 48 h on the expression level of P53. Each point is the mean ± SD of three independent experiments performed in duplicate. a: Significantly different from control group at *p* < *0.05* using ANOVA followed by Tukey-Kramer as post-hoc test.

Figure 4 shows the treatment of T47D cells with PFH-G9 (15 μg/ml) resulted in a significant decrease in p53 level by 58.5% compared to the control group. Additionally, TAM (15 μM) significantly decreased p53 level by 77.5% compared to the control group.

The primers sequences of MMP-2, MMP-9, caspase-3 and p53 are shown in Table (3).

**Table 3:** The primers sequences of MMP-2, MMP-9, caspase-3 and P53. Values were normalized to GAPDH and were expressed as relative expression levels.

Gene	Forward	Reverse
MMP-2	5-TGCCCAAGAATAGATGCTGAC-3	5-GAAAGGAGAAGAGCCTGAAGTG-3
MMP-9	5-CTTCT GCCCGGACCAAGGATAC-3	5-TTCAGGGCGAGGACCATAGAGG-3
P53	5-CCTCACCATCATCACACTGG-3	5-GCTCTCGGAACATCTCGAAG-3
GAPDH	5-TGAAGGTCGGAGTCAACGGATTT-3	5-GCCATGGAATTTGCCATGGGTGG-3

#### Effect of PFH-G9 on VEGF level in T47D cells

**Figure 5:**
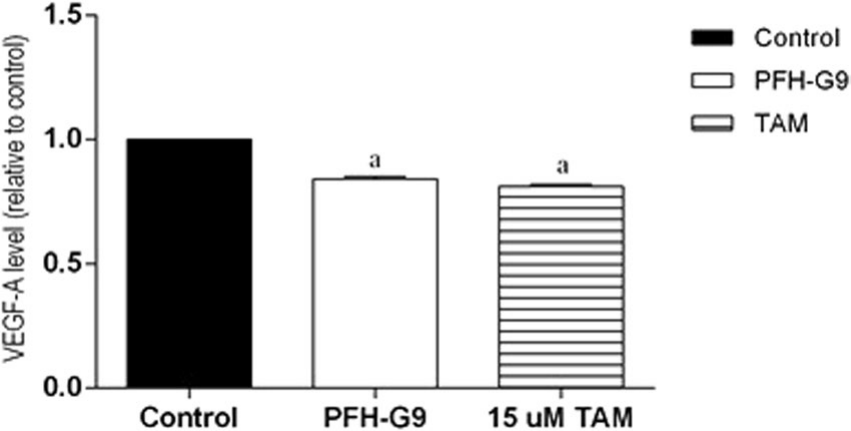
Effect of treatment of T47D cells with IC_50_ of PFH-G9 (15 μg/ml) or TAM (15 μM) for 48 h on VEGF level. Each point is the mean ± SD of three independent experiments performed in duplicate. a: Significantly different from control group at *p* < *0.05* using ANOVA followed by Tukey-Kramer as post-hoc test.

PFH-G9 and TAM resulted in a significant decrease in VEGF levels by 16% and 19% respectively compared to the control group Fig. 5.

### *In vivo* study

#### Effect on tumor volume

**Figure 6:**
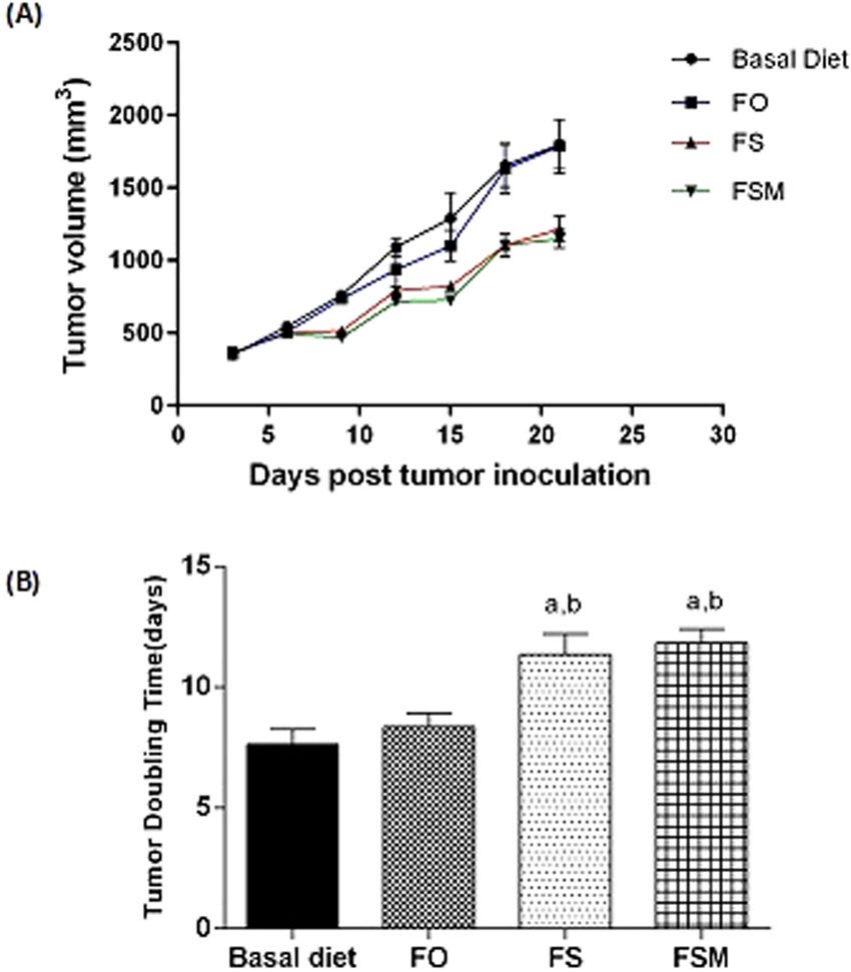
Effect of BD,FO, FS and FSM given as diet twice daily for three weeks in EAC-bearing female Swiss albino mice on (**A**) the mean tumor volume, (**B**) Tumor Doubling Time (TDT); the average days required by the tumor to reach double the initial tumor volume in EAC-bearing mice. Values were given as means ± SD (n = 7–12), a, b: Significantly different from BD and FO respectively at *p* < *0.05* using ANOVA followed by Tukey-Kramer as post-hoc test.

Flaxseeds (FS) and Flax meal (FSM) given as diet twice daily for three weeks to EAC-bearing mice were able to significantly retard tumor growth rate compared to the BD group Fig. 6. FS and FSM significantly increased the tumor doubling time (TDT; the average days required by the tumor to reach double the initial tumor volume) by 48.7% and 55%, respectively compared to the BD group. In contrast, flaxseed fixed oil (FSO) given as diet twice daily for three weeks to EAC-bearing mice didn’t induce any significant change on the tumor doubling time (TDT) compared to the BD feeding group.

#### Effect on GSH and MDA levels

**Figure 7:**
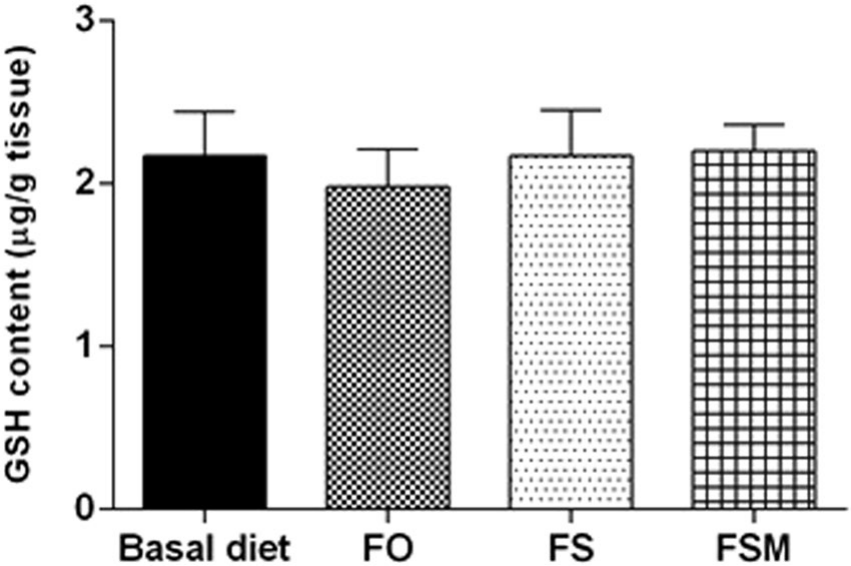
Effect of FO, FS and FSM given as diet twice daily for three weeks on GSH content in EAC-bearing mice. Values were given as means ± SD (n = 6).

**Figure 8:**
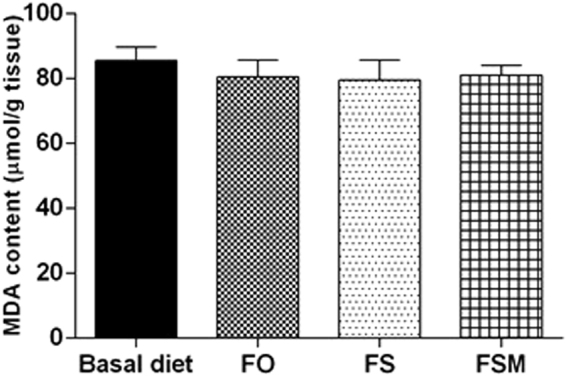
Effect of FO, FS and FSM given as diet twice daily for three weeks on MDA content in EAC-bearing mice. Values were given as means ± SD (n = 6).

As shown in Figs 7 and 8, FO, FS and FSM given as diet twice daily for three weeks to EAC-bearing mice didn’t result in any significant change in both GSH and MDA levels compared to EAC-bearing group given BD as diet twice daily for three weeks.

#### Effect on estrogen, Ki-67, insulin growth factor and progesterone expression, caspase-3, VEGF and MMP-2

Table (4) summarizes the results of biological markers; estrogen, Ki-67, insulin growth factor and progesterone measured in tumor specimens after the administration of FO, FS and FSM given as diet twice daily for three weeks to EAC-bearing mice have significantly decreased the expression of estrogen, Ki-67 and insulin growth factor. Moreover, FS and FSM significantly attenuated the expression of progesterone. Estrogen, Ki-67, insulin growth factor and progesterone-labelled cells were the least in FSM group.

**Table 4:** Effect of FO, FS and FSM given as diet twice daily for three weeks on estrogen receptor, Ki-67, insulin growth factor, progesterone, caspase-3, VEGF and MMP-2 expression in EAC-bearing mice.

Groups	Estrogen receptor expression (% of positively stained cells)	Ki-67 expression (% of positively stained cells)	Insulin growth factor expression (% of positively stained cells)	Progesterone expression (% of positively stained cells)	Caspase-3 expression (% of positively stained cells)	VEGF expression (% of positively stained cells)	MMP-2 expression (% of positively stained cells)
BD	47.38 ± 4.05	44.99 ± 2.56	41.91 ± 2.69	41.42 ± 2.73	9.01 ± 1.26	51.83 ± 1.94	88.83 ± 2.63
FO	35.44 ± 3.22^a^	34.67 ± 2.08^a^	35.02 ± 2.15 a	38.03 ± 3.84	26.11 ± 1.16^a^	25.02 ± 1.41^a^	87.50 ± 2.41
FS	20.59 ± 1.17^a,b^	21.41 ± 2.03^a,b^	21.61 ± 2.15^a,b^	19.601.86^a,b^	45.30 ± 1.86^a,b^	5.83 ± 1.47^a,b^	71.33 ± 1.96^a,b^
FSM	15.47 ± 2.00^a,b,c^	12.62 ± 1.00^a,b,c^	16.21 ± 2.32^a,b,c^	11.642.16^a,b,c^	76.16 ± 1.94^a,b,c^	6.01 ± 0.89^a,b^	51.83 ± 1.94^a,b,c^

Quantification of estrogen receptor, Ki-67, insulin growth factor, progesterone, caspase-3, VEGF and MMP-2 staining represent the percent of estrogen receptor, Ki-67, insulin growth factor and progesterone positive cells respectively per total number of cells in a field, across six fields. Values were given as means ± SD (n = 6), a, b and c: Significantly different from BD, FO and FS respectively at *p* < *0.05* using ANOVA followed by Tukey-Kramer as post-hoc test.

**Figure 9:**
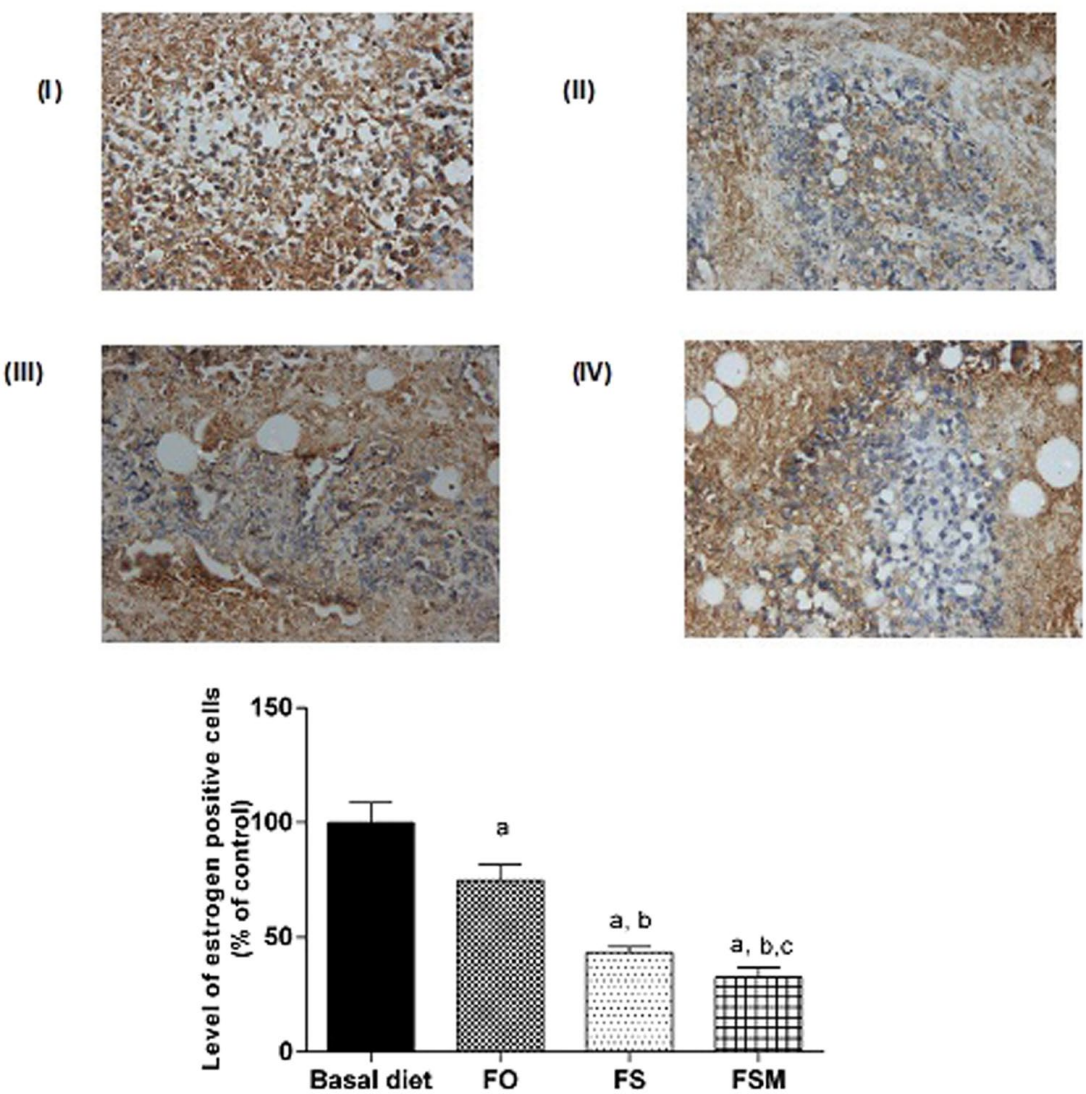
Effect of BD, FO, FS and FSM given as diet twice daily for three weeks on estrogen receptor expression in EAC-bearing mice. (**A**) Immunohistochemical staining of the marker; estrogen in EAC solid tumor sections (400×). BD(I), FO(II), FS(III) and FSM(IV). (**B**) Quantification of estrogen receptor immunopositive cells to the total area of the microscopic field across six fields. Values were given as means ± SD (n = 6), a,b and c: Significantly different from BD, FO and FS respectively at *p* < *0.05* using ANOVA followed by Tukey-Kramer as post-hoc test.

**Figure 10:**
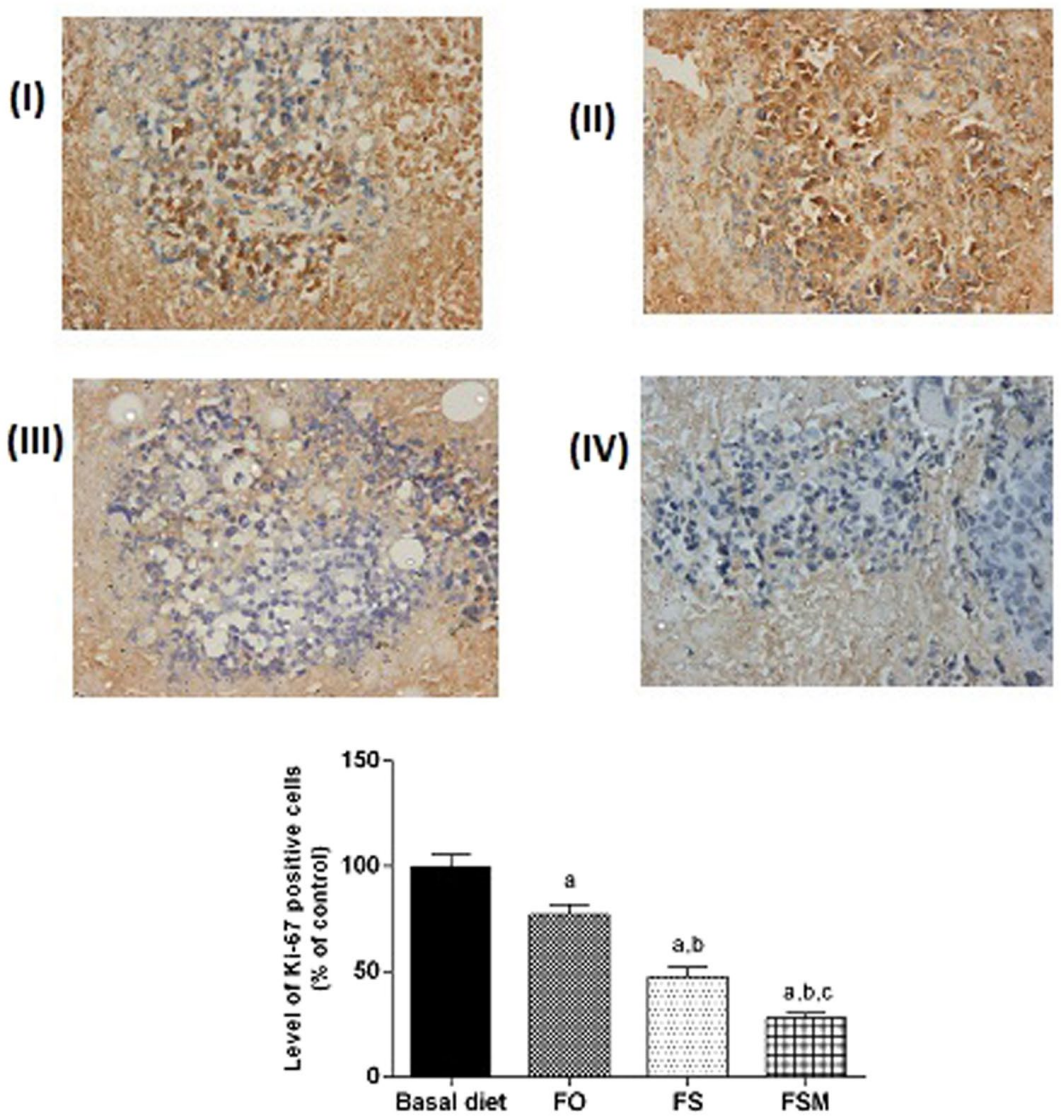
Effect of BD, FO, FS and FSM given as diet twice daily for three weeks on Ki-67 expression in EAC-bearing mice. (**A**) Immunohistochemical staining of the marker; Ki-67 in EAC solid tumor sections (400×) BD(I), FO(II), FS(III) and FSM(IV). (**B**) Quantification of estrogen immunopositive cells to the total area of the microscopic field across six fields. Values were given as means ± SD (n = 6), a,b and c: Significantly different from BD, FO and FS respectively at *p* < *0.05* using ANOVA followed by Tukey-Kramer as post-hoc test.

**Figure 11:**
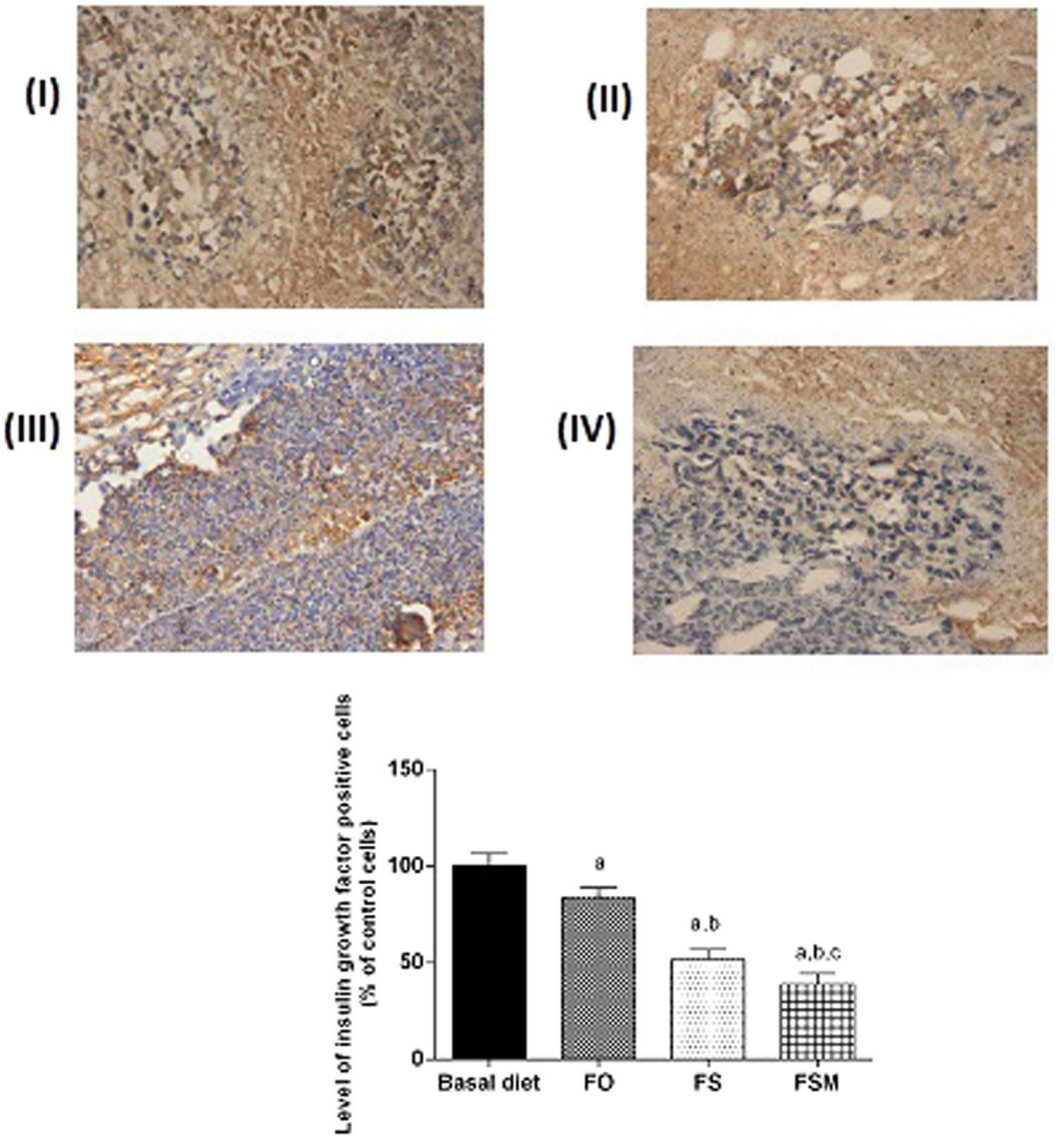
Effect of BD, FO, FS and FSM given as diet twice daily for three weeks on insulin growth factor expression in EAC-bearing mice. (**A**) Immunohistochemical staining of the marker; insulin growth factor in EAC solid tumor sections (400×) BD(I), FO(II), FS(III) and FSM(IV). (**B**) Quantification of estrogen immunopositive cells to the total area of the microscopic field across six fields. Values were given as means ± SD (n = 6), a,b and c: Significantly different from BD, FO and FS respectively at *p* < *0.05* using ANOVA followed by Tukey-Kramer as post-hoc test.

**Figure 12:**
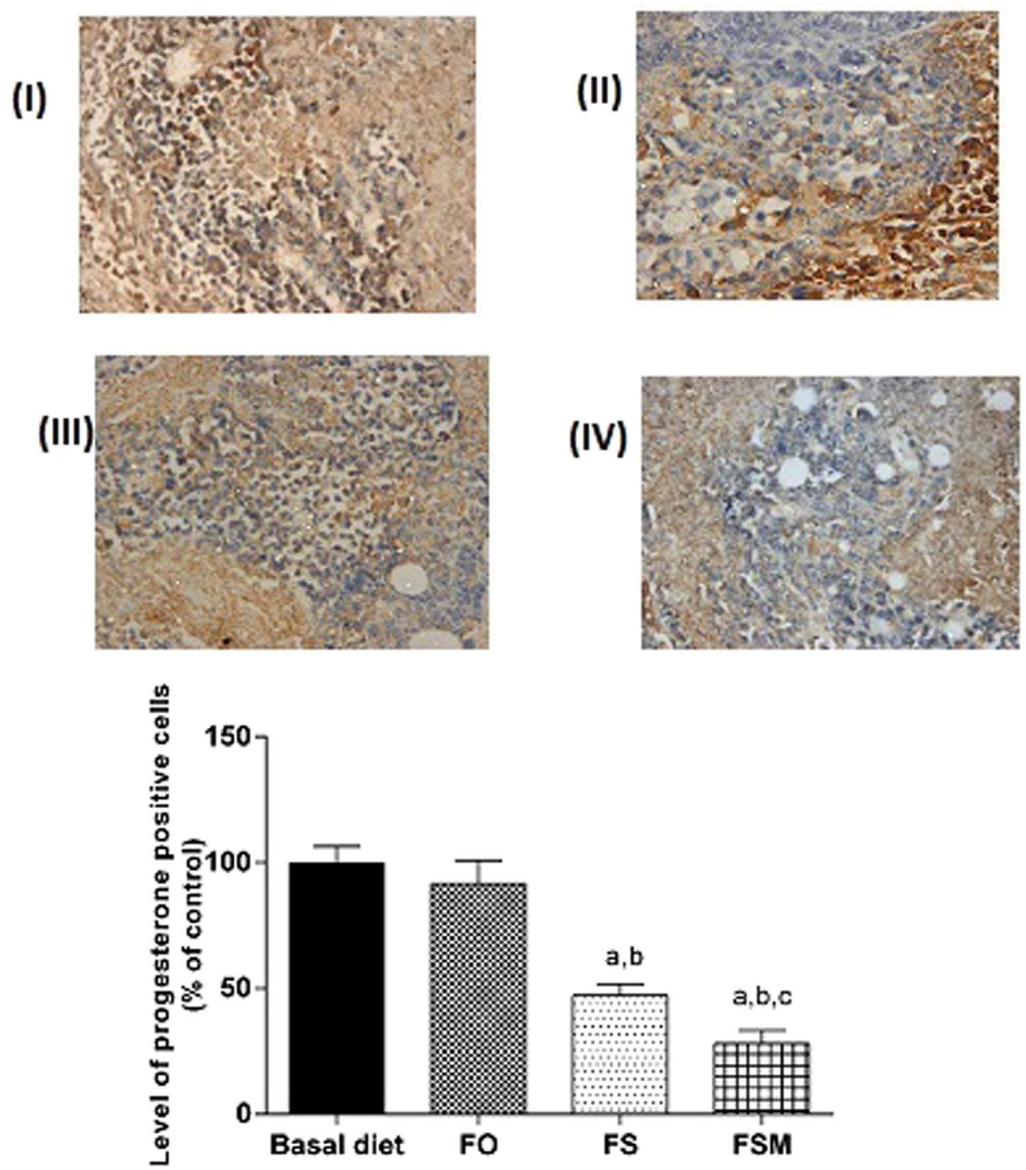
Effect of BD, FO, FS and FSM given as diet twice daily for three weeks on progesterone expression in EAC-bearing mice. (**A**) Immunohistochemical staining of the marker; progesterone in EAC solid tumor sections (400×). BD(I), FO(II), FS(III) and FSM(IV). (**B**) Quantification of estrogen immunopositive cells to the total area of the microscopic field across six fields. Values were given as means ± SD (n = 6), a, b and c: Significantly different from BD, FO and FS respectively at *p* < *0.05* using ANOVA followed by Tukey-Kramer as post-hoc test.

**Figure 13:**
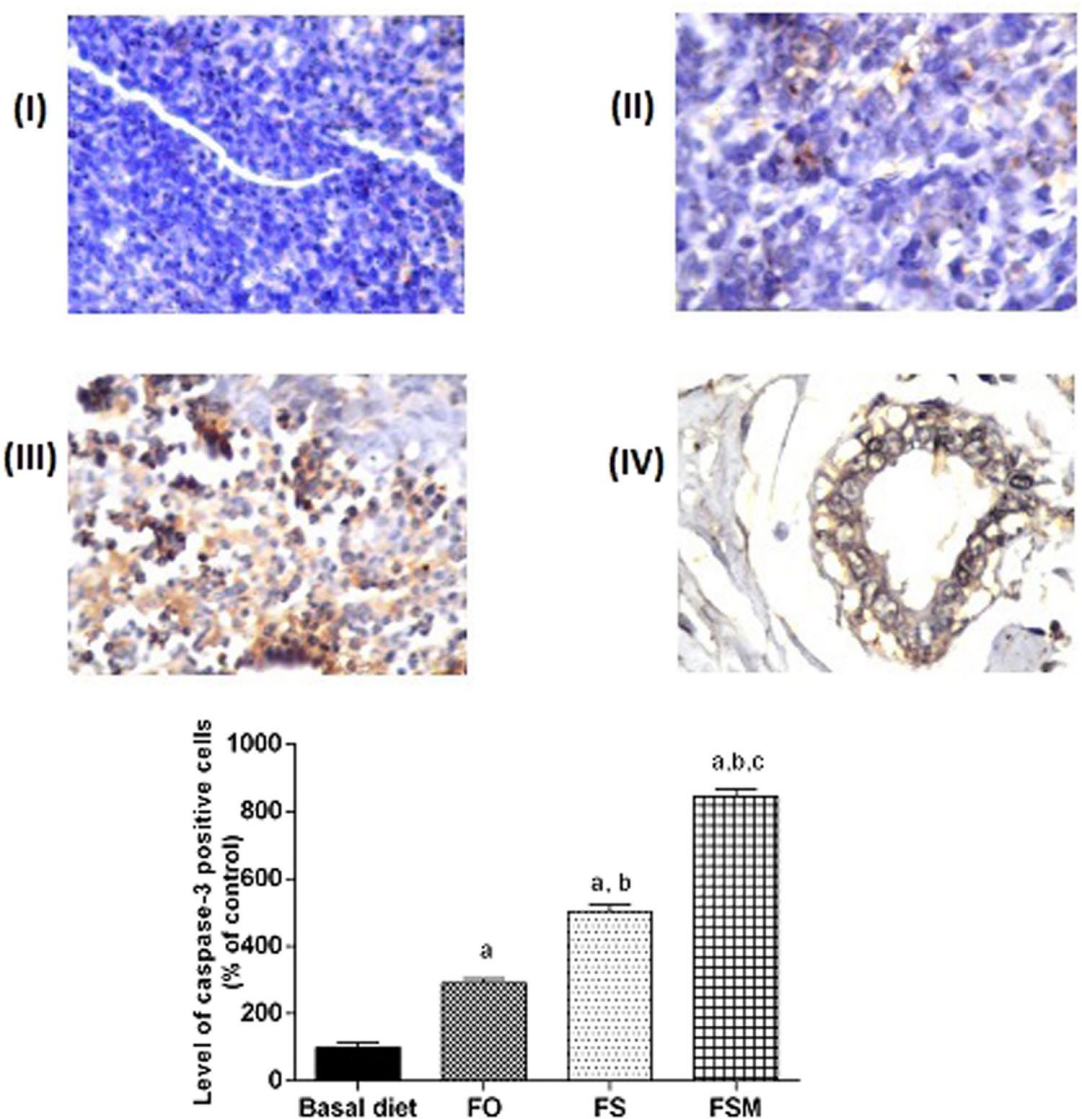
Effect of BD,FO, FS and FSM given as diet twice daily for three weeks on caspase-3 expression in EAC-bearing mice. (**A**) Immunohistochemical staining of caspase-3 in EAC solid tumor sections (160×). BD (I), FO (II), FS (III) and FSM (IV). (**B**) Quantification of caspase-3 immunopositive cells to the total area of the microscopic field across six fields. Values were given as means ± SD (n = 6), a, b and c: Significantly different from BD, FO and FS respectively at *p* < *0.05* using ANOVA followed by Tukey-Kramer as post-hoc test.

**Figure 14:**
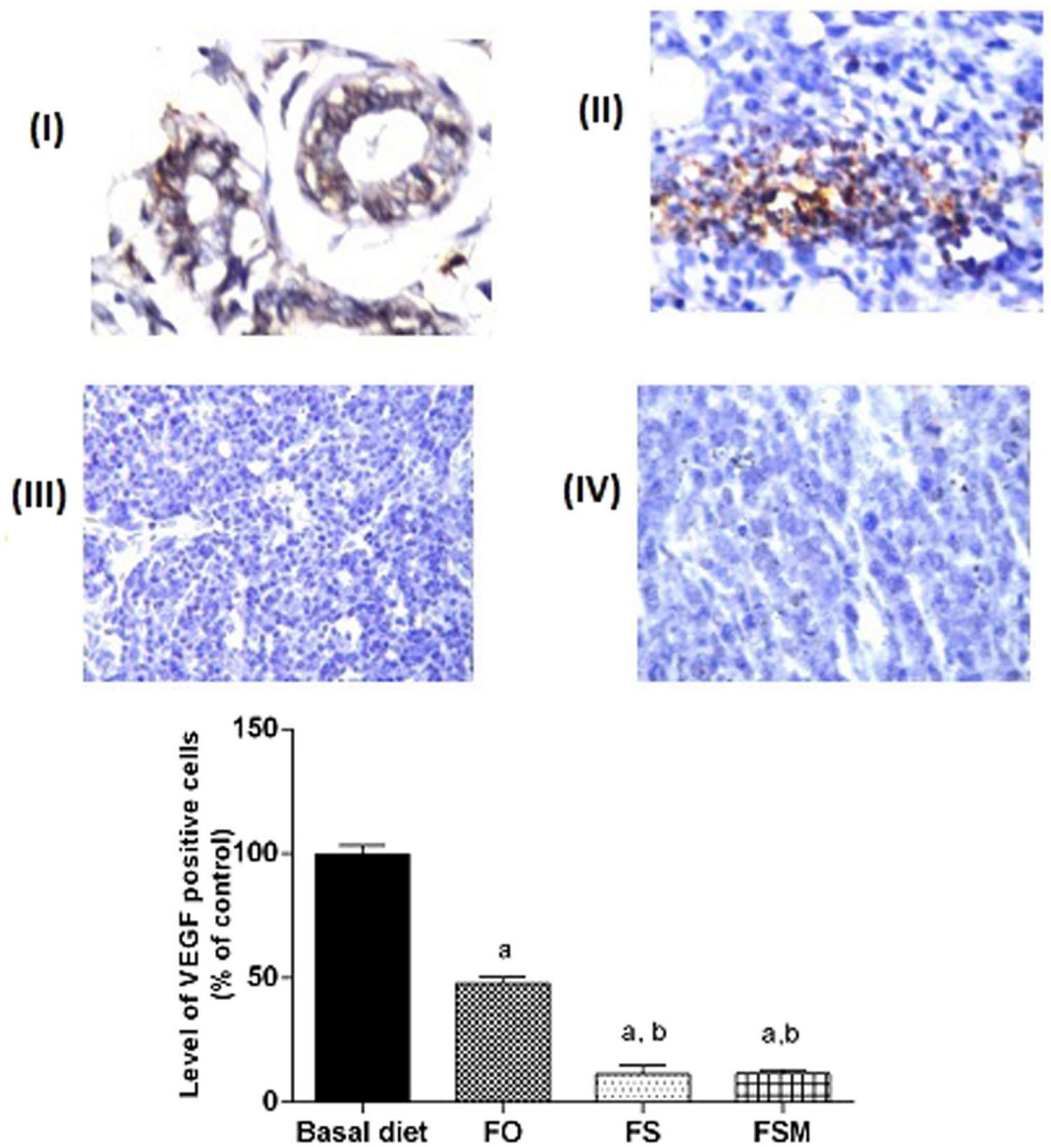
Effect of BD,FO, FS and FSM given as diet twice daily for three weeks on VEGF expression in EAC-bearing mice. (**A**) Immunohistochemical staining of VEGF in EAC solid tumor sections (160×). BD (I), FO (II), FS (III) and FSM (IV). (**B**) Quantification of VEGF immunopositive cells to the total area of the microscopic field across six fields. Values were given as means ± SD (n = 6), a,b and c: Significantly different from BD, FO and FS respectively at *p* < *0.05* using ANOVA followed by Tukey-Kramer as post-hoc test.

**Figure 15:**
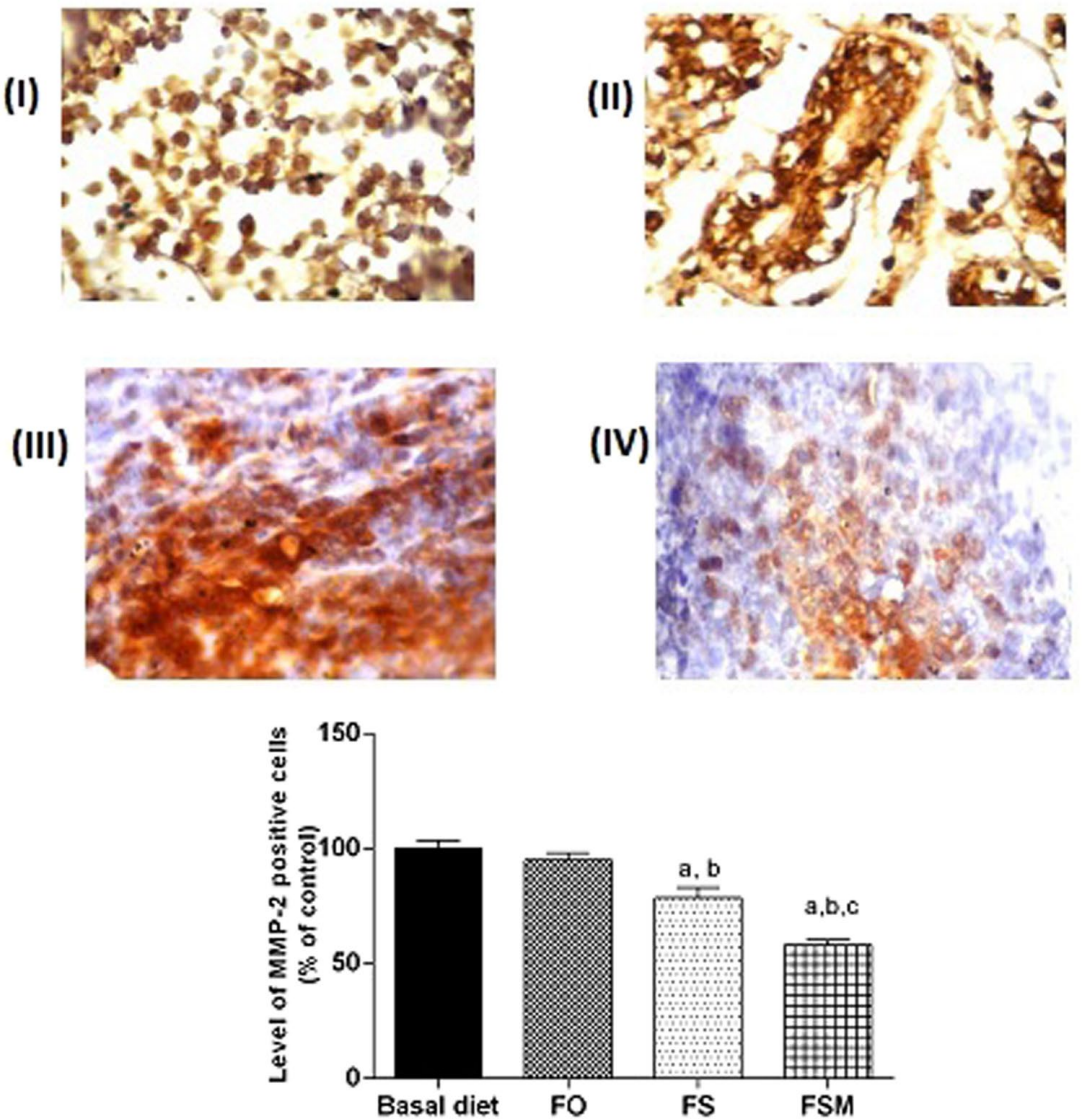
Effect of BD,FO, FS and FSM given as diet twice daily for three weeks on MMP-2 growth factor expression in EAC-bearing mice. (**A**) Immunohistochemical staining of MMP-2 in EAC solid tumor sections (160×). BD (I), FO (II), FS (III) and FSM (IV). (**B**) Quantification of MMP-2 immunopositive cells to the total area of the microscopic field across six fields. Values were given as means ± SD (n = 6), a, b and c: Significantly different from BD, FO and FS respectively at *p* < *0.05* using ANOVA followed by Tukey-Kramer as post-hoc test.

Regarding estrogen, its expression was reduced by 26, 56 and 67% in FO, FS and FSM respectively compared to the BD group (Fig. 9). Moreover, FO, FS and FSM diets significantly repressed the expression of Ki-67 by 23, 52 and 72%, respectively compared to the BD group (Fig. 10). Also, the expression of insulin growth factor was significantly decreased after the administration of FO, FS and FSM as diets by 16, 48 and 61% respectively compared to BD group (Fig. 11). Additionally, progesterone expression was significantly suppressed by 53 and 72% in animals fed FS and FSM diets respectively compared to the BD group (Fig. 12). Interestingly, FO, FS and FSM diets significantly enhanced caspase-3 expression by 192%, 404% and 746%, respectively, compared to the BD group (Fig. 13). Concerning VEGF, its expression was attenuated by 52%, 89% and 88% respectively, compared to the BD group (Fig. 14). Moreover, FS and FSM diets significantly mitigated the expression of mmp-2 by 22 and 42% respectively compared to the BD group (Fig. 15).

## Discussion

The purified hydrolysates from different cultivars of Flaxseed were tested against three different breast cancer cell lines, two ER + (MCF-7& T47D) and one triple negative (MDA-MB231), in addition to a colorectal (HCT-116) and cervix (HELA) cancer cell lines. Moreover, the cytotoxicity of the extracted hydrolysates were tested against normal cells (HFB4) to ensure their safety. According to the plant screening program of the US National Cancer Institute, for a crude extract to have *in vitro* cytotoxic activity, its IC_50_ value should be less than 20 µg/ml, at incubation period of 48 and 72 hours^18^. On this basis, PFH-G9 hydrolysate showed the most effective cytotoxic activity against HELA, MCF7 and T47D cell lines with IC_50_ 17.4, 13.8 and 15.8 µg/ml, respectively. The high anticancer activity of PFH-G9 could be attributed to its content of SDG as the HPLC analysis had shown that PFH-G9 contains the highest concentration of SDG (81.64 mg/g) when compared to the other cultivars. A very critical stage in cancer prognosis is the development of tumor cell invasion and metastasis that is responsible for the mortality and morbidity of most cancer patients. Matrix metalloproteinases (MMPs) have a substantial role in the classic hallmarks of cancer including tumor growth and the multistep processes of invasion and metastasis^19^. MMP-2 and MMP-9 were investigated as one of the possible anticancer mechanisms of flaxseed. Our results showed that PFH-G9 significantly attenuated MMP-2 and MMP-9 levels in T47D cells although, TAM an estrogen receptor modulator used in ER positive breast cancer patients treatment significantly enhanced MMP-2 and MMP-9 levels in T47D cells. Similar upregulation of MMP2 and 9 by TAM in T47D cells was previously reported^20^. The decreased levels of MMP-2 and 9 in this study cope well with a previous study which revealed that enterolactone, a mammalian lignan derived from flaxseed down-regulated the metastasis-related MMP-2, MMP-9 and MMP-14 gene expressions in MCF-7 and MDA-MB231 cell lines^2^. In addition, it was reported that administration of TAM together with a physiologic level of estradiol to nude mice bearing breast cancer tumors (MCF7) caused an increase in endostatin, MMP-2 and MMP-9 levels compared with estradiol treatment only^21^. However, it was found the proliferation of ER beta-positive colon cancer cell line (HT-29) was inhibited by TAM only in high concentrations where it could bind to ER beta to down-regulate the expression of MMP-7^22^.

VEGF has a pivotal role in regulation of angiogenesis, a physiological process which is very essential to survival of cancer cells, together with cell proliferation and migration^23^. Cancer usually enhances VEGF expression, increasing its concentration due to tumor aggression and the poor prognosis, hence VEGF is involved in cancer pathology^24^. In the present study, significant decrease in VEGF expression was detected after treatments with PFH-G9 and TAM. These data are in harmony with a previous study which revealed that flaxseed and its lignans enterodiol and enterolactone have the potential to decrease angiogenesis *in vitro* and *in vivo* via reducing the estradiol-induced VEGF secretion in MCF-7 cells and tumor explants, respectively^25^.

Apoptosis (or Programmed Cell Death Type I) is as a safeguard system preventing metastasis at all crucial steps and preventing cancer progression^26^. Apoptosis is a caspase-dependent process which has a direct effect on the development, differentiation and proliferation of cancer. Many researches demonstrated that caspase 3, the executioner caspase, has a vital role in controlling the cell destiny, through the regulation of apoptosis, it was screened to ascertain the oncolytic activity of PFH-G9 in T47D cells. Caspase-3 significantly increased by treatment with either PFH-G9 or TAM^27^. P53 is the most pivotal tumor suppressor gene encoding a sequence-specific transcriptional regulator that controls a plethora of biological functions, including cell-cycle progression, senescence, differentiation, DNA repair, and apoptosis^28^. Normal p53 function was shown to be crucial in the induction of apoptosis in human and murine cells following DNA damage^25^. P53 is the most commonly mutated tumor-suppressor gene in human cancers. The possible role of p53 in the induction of apoptosis by PFH-G9 was investigated. Our data manifested that both PFH-G9 and TAM significantly decreased p53 levels in T47D that expresses mutant p53 in T47D of breast cancer cell line that led to massive apoptosis. Contrary to our results, a previous study showed that flaxseed treatment significantly upregulated p53 mRNA in MCF-7 and MDA-MB-231 cells, thereby inducing apoptosis^29^. The reduction of P53 expression in flaxseed- treated T47D while upregulation of expression in MCF7 treated cells is advantageous for both cell lines as T47D cells has mutant P53 and MCF7 has wild type P53. These data are in agreement with those reported before, which stated that mutant p53 silencing which specifically targets mutant p53 in T47D cells induced massive apoptosis as evidenced by morphological cell blebbing, PARP cleavage, and annexin V/PI staining, however, apoptosis was not observed in MCF-7 or MCF-10A cells that express only wild type^20,30^.

The study was further extended to study the oncolytic effect of different flaxseed diet modalities FO, FS, and FSM diets in EAC-bearing mice. FS and FSM were found to repress the tumor growth. Moreover, all flaxseed diets; FO, FS, and FSM significantly reduced the expression of Ki-67; a nuclear protein expressed in proliferated cells and is used as cell proliferation marker^31^. Many studies have asserted the antitumor activity of dietary flaxseed^32–34^. Our data revealed that the three dietary formulation significantly reduced the expression of IGF-1; a tyrosine kinase known to be mitogenic, and playing a crucial role in cancer development^35^. In agreement with our results, Chen *et al*.^11^ have shown that flaxseed reduced the developed human breast cancer growth and metastasis in a nude mice model, and this effect is partly due to its down-regulation of insulin-like growth factor I and epidermal growth factor receptor expression. Additionally, flaxseed oil was found to reduce MCF-7 tumor growth partially through IGF-1 signaling pathway^36^. The percentage reduction in tumor growth correlated well with estrogen and progesterone status, where FS and FSM significantly repressed the expression of estrogen and progesterone, while FO on reduced the expression of estrogen. A previous study has shown that FS reduced human breast tumor growth (MCF-7) in athymic mice through the downregulation of ER- and growth factor- mediated cell signaling^37^. Moreover, an early study revealed that flaxseed extracts significantly decreased the production of progesterone in jeg3 tumor cell line^7^. In addition, the *in vitro* results regarding caspase-3, VEGF and MMP -2 were further confirmed *in vivo*, where FO, FS and FSM significantly enhanced caspase-3 expression, while they significantly mitigated VEGF and MMP-2 expression.

In conclusion, our results have asserted the antitumor activity of Egyptian flaxseed especially Giza-9 and its dietary formulations that involved apoptotic, antiangiogenic and antimetastatic effects.

## Materials and Methods

### Chemicals

RPMI-1640 Medium, fetal bovine serum, dimethylsulfoxide (DMSO), Ellman’s reagent [5,5-Dithio-bis-(2-nitro bezoic acid)], β-mercaptoethanol, glutathione (GSH), glutathione reductase, sodium dodecyl sulfate (SDS), sodium bicarbonate, 1,1.3,3-tetramethoxypropane, trichloroacetic acid (TCA) and thiobarbituric acid were all purchased from Sigma Aldrich Chemical Co. (St. Louis, MO, USA). Secoisolariciresinol diglucoside (SDG) for HPLC analysis was purchased from ChromaDex (Santa Ana, CA, USA). Triton X-100, penicillin, streptomycin were procured from MP Biochemical (Santa Ana, California, USA). All other chemical reagents and extraction solvents were of analytical grade, and all analysis solvents were of HPLC grade and they were obtained from E-Merck. Tamoxifen (TAM) was obtained from Sigma Aldrich Chemical Co. (St. Louis, MO, USA). Each vial of TAM contains one gm white powder.

### Plant Material

Seeds of six *L. usitatisimum* L. cultivars, were collected in winter 2012/2013 from different localities in Egypt *viz*. Qaliubiya, Sharkia, Gahrbia and Kafr El-Shakh and Giza Governorates, and identified by staff members of Fiber Crops Research Institute, Giza. Cairo, Egypt. The six flaxseed cultivars were identified namely; Sahka 1, Sakha 2, Sakha 3, Sakha 4, Giza 9 and Giza 10.

### Cell Lines

Three human breast carcinoma cell lines; MCF-7, T47D, and MDA-MB231, together with colon carcinoma cell line (HCT-116), cervix cell line (HELA) and normal fibroblast cell line (HFB4) were used in this study. They were obtained frozen in liquid nitrogen (−80 °C) from ATCC (MO, USA). The tumor cell lines were maintained by serial sub-culturing at the National Cancer Institute, Cairo, Egypt in RPMI-1640 medium supplemented with 10% fetal bovine serum, 100 U/ml penicillin, 100 mg/ml streptomycin, and 3 mM/l glutamine. The cells were trypsinized every 3 days. They were cultured in a humidified incubator supplied with 5% CO_2_ and temperature 37 °C.

### Animals

Female Swiss albino mice weighing 20–25 g, were obtained from the animal house of National Cancer Institute, Cairo, Egypt. All of the animal handling and study procedures were approved by the research ethics committee of Faculty of Pharmacy, Cairo University, Cairo, Egypt (PI 1563), and was conducted with the “Guide for the Care and Use of Laboratory Animals”. Animals were kept under standard laboratory conditions of temperature and humidity. They were provided with standard chow and water and housed in plastic cages. For the assessment of the antitumor activity *in vivo*, mice-bearing solid Ehrlich carcinoma (EAC) cells (2 × 10^6^) were transplanted subcutaneously in the right thigh of the lower limb mice.

### Preparation of purified flaxseed hydrolysates

The seeds of each of the six cultivars of flaxseed (50 gm) were separately crushed in a mill, followed by defatting with *n*-hexane (3 × 400 ml), at 60 °C, using ultrasonic bath, for 30 min. The defatted powder was then extracted with MeOH (80%) at 60 °C for 1hr (3 × 400 ml). The resulting methanolic extract was filtered, pooled and concentrated under vacuum (at 60 °C).

The methanolic extract in each case was hydrolyzed using NaOH (0.5N) for 3 hr. at room temperature. The hydrolysate solution in each case was then neutralized with acetic acid (0.5N). The neutralized hydrolysates were separately purified on Diaion HP-20 column using water and methanol, as eluents. The methanol fraction was evaporated till dryness and used for HPLC analysis and screening of cytotoxic activity.

### Sample preparation for HPLC analysis

About twenty milligram of each of the purified methanol fraction was accurately weighed, transferred to 10 ml volumetric flask, and completed with methanol to 10 ml. The solution was then filtered through 0.45 µm membrane filter and subjected to HPLC analysis.

### HPLC analysis

HPLC was used to determine the richest hydrolysate in secoisolariciresinol diglucoside (SDG) in each of the 6 cultivars. HPLC analysis was carried out using an Agilent 1100 series HPLC system (Agilent Technologies, Palo Alto, CA), equipped with a quaternary pump G1311A, degasser G1322A, UV detector and an Agilent ChemStations software. Separation was carried out on a Lichrosphre C-18 column (250 × 4.6 mm, 5 µm) (Merck, Germany). The mobile phase consisted of acetonitrile (solvent A) and 0.3% phosphoric acid (solvent B), using a gradient elution program, as follows: from 11% A/B to 25%A/B in 15 min, from 25% A/B to 60% A/B in 10 min, from 60% to 80% A/B in **5** min and from 80%A/B back to 11% A/B in 5 min. The flow rate was 0.9 ml min.^−1^. Injection volume was 20 µl. Analysis was carried out at room temperature and the detection wavelength was set at 280 nm.

### Preparation of standard solution

Standard stock solution of pure secoisolariciresinol diglucoside (SDG) was prepared by dissolving 1.0 mg in 10 ml methanol in a volumetric flask. This stock solution was serially diluted to prepare five different concentrations (125, 250, 500, 1000, 2000 µg/ml), which were injected in triplicates for establishing the calibration curve (Fig. 1A).

## Biological Assays

### Screening of *in vitro* cytotoxicity of the purified flaxseed hydrolysates (PFH)

#### Cytotoxicity assay

The PFH samples from the 6 cultivar were subjected to screening for antitumor activity on 5 cancer cell lines; three human breast carcinoma cell lines; MCF-7, T47D, and MDA-MB231, together with colon carcinoma cell line (HCT-116), cervix cell line (HELA) and normal fibroblast cell line (HFB4) using the sulforhodamine B (SRB) colorimetric assay^38^. Each PFH sample was dissolved in DMSO and tested at concentrations of 1, 5, 10, 20 and 50 μg/ml. Tamoxifen (TAM) was used as a positive control at the same concentration range. Final concentration of DMSO in the culture medium was maintained at 0.1% to avoid solvent toxicity. Cells were seeded in 96 well plates at a density of (2 × 10^3^ to 2 × 10^5^cells/well) in RPMI-1640 supplemented medium for 24 h, and then incubated with different concentrations of hydrolysate for 48 hrs. Cells were fixed with 50% trichloroacetic acid for 1 h at 4 °C. Wells were washed thrice with water, stained for 30 min at room temperature with 0.4% SRB dissolved in 1% acetic acid and then washed four times with 1% acetic acid. The plates were air dried and the dye was solubilized with 100 μl/well of 10 mM tris base (pH 10.5) for 5 min on a shaker at 1600 rpm. The optical density (OD) of each well was measured spectrophotometrically at 570 nm with an ELIZA microplate reader (Meter tech. S960, USA). The IC_50_ values were calculated (Graph Pad, Prizm ISI® software, Version 5).

Cytotoxicity was expressed as the percent of viable cells relative to cells incubated in the presence of 0.1% DMSO solvent control. The concentration of the PFH inhibiting cell growth by 50% relative to solvent control (IC_50_ value) was also determined. Each measurement was performed in triplicate.

#### RNA Purification and Real-Time polymerase chain reaction (qPCR) Analysis

To study the effect of treatment of T47D cell line with 15.9 µg/ml of PFH-G9 or TAM (15 µM) on the expression levels of metastatic markers, matrix metalloprotinases 2 and 9 were determined using ***qPCR***. Cells were collected from both the control and the treated and total RNA was extracted following the protocol of the RNeasy Mini Kit (Qiagen, Valencia, CA, USA). Reverse transcription was completed using High capacity cDNA archive kit (Applied Biosystem, California,USA), then real time PCR was performed in triplicate on an ABI 7500 Fast Real-Time PCR System using the GoTaq PCR master mix (Promega, Madison, U.S.A) for detection of the expression of 1-α,metalloproteinase MMP-2 and MMP-9 and p53on a 7900HT fast Real-Time PCR System (Applied Biosystems, California, USA). Fast amplification parameters were as follows: one cycle at 95 °C for 10 min, followed by 40 cycles at 95 °C for 15 s, and 60 °C for 1 min. All primers were purchased from Invitrogen (California,USA). Quantitative analysis of data was performed by using the∆∆ Ct method^39^. Values were normalized to GAPDH and were expressed as relative expression levels.

#### Assay of Caspase-3 activity

T47D cells were cultured in 75 cm^3^ flasks, left till 70–80% confluent, cells were treated with PFH-G9 (15.8 µg/ml) or TAM (15 µM) and incubated for 48 h. The treated and control cells were lysed in a RIPA lysis buffer containing protease inhibitors. The Caspase-3 activity was assessed following spectrophotometric method at 450 nm in lysate using ELISA kit (Invitrogen, **Carlsbad, CA**,USA) following the manufacturer’s instructions^40^. Each concentration repeated two times and the experiment was carried out three independent times. The activity was calculated relative to the corresponding protein content.

#### Assay of vascular endothelial growth factor (VEGF-A) level

VEGF was determined in cell culture medium using eBioscience ELISA kit (San Diego, CA, USA). Cells were plated in 6 well plates with 5*10^4^. After treatment with PFH-G9 (15.8 µg/ml) or TAM (15 µM), the medium was aspirated, centrifuged at 10,000 rpm for 10 min at 4 °C to remove any dead cells. The clear supernatant was used for assay following the manufacturer’s instructions^41^.

#### Protein concentration assay

Protein concentrations were measured in the medium and cell lysate using protein kit (Pierce, Rockford, IL, USA)^42^. The method depends on the binding of Comassie Brilliant Blue G-250 dye with protein and forming a complex which can be measured spectrophotometrically at 595 nm then the concentration was determined using a standard calibration curve.

### *In vivo* experiments

#### *In vivo* anticancer activity of Giza 9 cultivar

Forty animals were divided in to 4 groups, each consists of 10 animals. Group I: animals of this group were fed with normal pellets of Basal Diet (BD) and served as control. Group II: animals of this group were fed normal pellets BD containing 10% Flaxseed (FS). Group III: animals of this group were fed on normal pellets containing 10% Flaxseed meal (FSM). Finally, Group IV: animals of this group were fed pellets mixed with 10% Flaxseed oil (FSO). The diet was supplied twice a day for three weeks. The change in weight of the animals of different groups was monitored twice weekly for three weeks

#### Antitumor effect

The change in tumor volume was measured every other day using venire caliber and calculated by the following formula, Tumor volume mm^3^ = 0.52 A^2^ × B, where A and B denote the minor & major tumor axis, respectively. At the end of the experiment the animals were sacrificed and the tumors were isolated, excised and weighed^43^.

#### Determination of reduced glutathione (rGSH) and MDA contents in solid tumor

After the last treatment, animals were anesthetized. Tumors were quickly excised, washed with saline, blotted with a piece of filter paper, and homogenized using a Branson sonifier (250, VWR Scientific, Danbury, Connecticut, USA). The homogenates were centrifuged at 800 g for 5 min at 4 °C to separate the nuclear debris, then supernatant was again centrifuged at 10,500 g for 20 min at 4 °C. Levels of glutathione and MDA were determined as previously described by (Ellman 1959)^44^ and (Draper and Hadley 1990)^45^, respectively.

#### Immunohistochemical staining (IHC) of estrogen, androgen receptors and KI67 caspase-3, VEGF and MMP-2

Representative tissue samples were fixed in 10% neutral phosphate-buffered formalin, embedded in paraffin, and sectioned at 5 µm thickness. Sections were incubated with mouse monoclonal anti-estrogen receptors, progestrone receptors, insulin like growth factor, KI 67, **caspase-3, VEGF and MMP-2** (Sigma Aldrich Chemical Co., USA) as a primary antibody at a dilution of 1:150 overnight at 4 °C. After rinsing three times, sections were incubated with polymer horseradish peroxidase HRP secondary antibody (Sigma Aldrich Chemical Co., USA) for 1 hr. Immuno-reactivity was detected by the standard avidin–biotin immunoperoxidase method.

### Statistical analysis

All data were expressed as mean ± S.D. The difference between the treated samples and the untreated controls was analyzed by one way ANOVA followed by Tukey multiple comparison test in which p < 0.05 was considered as significant. All statistical analysis was performed using GraphPad In Stat, version 5.0 (GraphPad, San Diego, California, USA). Statistical significance was set at p < 0.05.
